# Recent Trends in the Use of Electrical Neuromodulation in Parkinson’s Disease

**DOI:** 10.1007/s40473-018-0154-9

**Published:** 2018-04-24

**Authors:** John-Stuart Brittain, Hayriye Cagnan

**Affiliations:** 1 School of Psychology, University of Birmingham, Edgbaston, Birmingham, B15 2TT UK; 2 Institute of Neurology, University College London, London, UK

**Keywords:** Parkinson’s disease (PD), Deep brain stimulation (DBS), Non-invasive transcranial brain stimulation (NTBS), Transcranial direct current stimulation (tDCS), Transcranial alternating current stimulation (tACS), Direct cortical stimulation

## Abstract

**Purpose of Review:**

This review aims to survey recent trends in electrical forms of neuromodulation, with a specific application to Parkinson’s disease (PD). Emerging trends are identified, highlighting synergies in state-of-the-art neuromodulation strategies, with directions for future improvements in stimulation efficacy suggested.

**Recent Findings:**

Deep brain stimulation remains the most common and effective form of electrical stimulation for the treatment of PD. Evidence suggests that transcranial direct current stimulation (tDCS) most likely impacts the motor symptoms of the disease, with the most prominent results relating to rehabilitation. However, utility is limited due to its weak effects and high variability, with medication state a key confound for efficacy level. Recent innovations in transcranial alternating current stimulation (tACS) offer new areas for investigation.

**Summary:**

Our understanding of the mechanistic foundations of electrical current stimulation is advancing and as it does so, trends emerge which steer future clinical trials towards greater efficacy.

## Introduction

As we pass the bicentennial of James Parkinson’s seminal essay on the shaking palsy [1], it is fitting to acknowledge that great strides have been made in our understanding and management of this common neurological condition (2). While many approaches to neuromodulation have been trialled in the treatment of Parkinson’s disease (PD) over the years, the most striking is undoubtedly the advent of levodopa as a front-line treatment. The effectiveness of this pharmacological manipulation is remarkable, yet leads to gross motor complications including devastating levodopa-induced dyskinesias (for a review, see [3]). In 1990, it was observed that lesioning the subthalamic nucleus (STN) reversed experimental Parkinsonism [4]. This seminal observation was followed by the first trial of deep brain stimulation (DBS) of the STN in 1994 as a treatment for PD [5]. The procedure proved remarkably effective at ablating both the hyper- and hypo-kinetic symptoms of the condition. DBS has since become a common treatment for a range of disorders, with over 100,000 people having undergone implantation worldwide [6]. More recently, transcranial current stimulation (tCS) has re-emerged as a method to non-invasively modulate brain activity and has been trialled in a variety of guises to treat a number of neurological disorders, including PD.

This review will focus on recent trends in the therapeutic application of electrical forms of neuromodulation, with a specific focus on PD. We briefly survey the use of DBS, transcranial direct current stimulation (tDCS) and transcranial alternating current stimulation (tACS) with application to PD, drawing parallels and highlighting differences between these approaches where appropriate. Direct (invasive) cortical stimulation is considered, with perspectives for future applications. Electroconvulsive therapy (ECT) is not directly discussed, but recent reviews and perspectives are available [7, 8]. Common goals and cross-modal perspectives are given, regarding emerging neuromodulation strategies. The focus on electrical forms of neuromodulation will necessarily omit a wealth of recent technological developments and innovations which remain outside the scope of this review; notably MR-guided ultrasound [9, 10] which has garnered much recent attention for achieving focussed unilateral sub-cortical lesions. For an overview of PD pathology, management and treatment, the reader is directed towards the excellent bicentennial essay of Obeso et al. [2].

While the various forms of stimulation considered here involve the application of electrical currents, the approaches and mechanism of action of DBS, tDCS and tACS are quite distinct and will necessitate tailored strategies in therapeutic or rehabilitative applications. DBS is an invasive neurosurgical procedure where electrical impulses are delivered to precise, stereotactically targeted regions of the brain. tDCS meanwhile involves the application of broad non-invasive transcranial electric fields that modulate cortical excitability in a stimulation polarity dependent fashion [11]. tACS operates principally through sub-threshold electrical modulation of the membrane potential and is hypothesised to induce synchronous states [12]. Yet despite these seemingly disparate approaches, with distinct mechanistic underpinnings, all have been trialled to treat PD and share common traits. One striking similarity is the push towards patient-centric, tailored forms of therapy that include complex field steering and the advent of on-demand and closed-loop strategies. Here, we review recent advances in their respective fields, drawing parallels where appropriate to guide future efforts with the ultimate goal of focussing investigations towards greater clinical utility.

## Deep Brain Stimulation

DBS has proven remarkably effective at alleviating both the hypo- and hyper-kinetic symptoms of PD [13, 14], achieving its therapeutic benefit through the delivery of brief high-frequency (typically in excess of 100 Hz) electrical impulses to targeted nuclei through electrodes implanted in sub-cortical regions of the brain that would otherwise remain inaccessible to intervention. Indeed, DBS remains the most prevalent neurosurgical procedure for PD [15], providing effective relief of motor and some non-motor symptoms [16]. However, as previously noted [17], DBS hardware has remained largely static for many years, likely a victim of its own success. Yet a raft of recent innovations stand ready to allow tailored forms of stimulation that may alleviate unwanted side-effects, or even boost the efficacy of stimulation itself. While the exact mechanism of action of DBS still remains unknown, it is now believed that DBS efficacy is rooted in a reversible information lesion that disrupts the expression of pathological neural activity across the motor circuit [18]. In the case of PD, the classic electrophysiological hallmark in the basal ganglia is elevated beta (13–30 Hz) activity [19], which is suppressed by dopamine and high-frequency DBS [20–24].

### Field Shaping

Although DBS delivers a focal form of stimulation with electrodes entrenched directly in neural tissue, the distribution of the induced electric field is ultimately reliant upon the geometry of the electrode contacts. Traditionally these have been cylindrical, producing an omnidirectional field. However, it has long been known that stimulation of adjacent structures can lead to unwanted side-effects including, most commonly, parasthesias and dysarthria [25]. Recently, the first reports on the clinical efficacy of electric field shaping were published, a technique achieved through the implantation of next-generation electrodes that possess either segmented electrodes (typically breaking each cylindrical contact into three radial aspects), or more generic multi-contact designs, permitting the formation of complex stimulation fields.

The first double-blind study into the efficacy of directional DBS electrodes found that the preferred orientation for stimulation significantly improved the therapeutic window over alternate directions, and over omnidirectional stimulation [26]—a result which has since been confirmed [27–32]. The therapeutic window not only takes into account the suppression of motor symptoms but also considers the onset of stimulation-induced side effects. However, the added complexity of programming segmented electrodes emphasises the need for electrophysiological biomarkers to guide contact selection, and perhaps even tune stimulation parameters. Spectral analysis of local field potentials (LFPs) from directional electrodes have already identified localised spectral signatures of beta (and tremor) activity [30, 31, 33] and have directly related stimulation at those contacts to broader therapeutic windows [30, 31].

### Adaptive Stimulation

DBS therefore provides a highly focal, if functionally non-specific form of neuromodulation. Essentially, stimulation is not reactive to the current brain state [34]; although this does not imply that the effects of stimulation are not brain-state dependent, as elevated levels of beta and tremor-related signals appear more strongly modulated than similar activity occurring at lower intensities, or activity residing in other frequency bands [35]. Despite this, while high-frequency pulse trains are effective in improving the motor (and some non-motor) aspects of the disease, DBS can produce unwanted behaviours such as impulsivity [36], parasthesias, dysarthria, postural instabilities, and even weight gain [37]. It has been proposed that on-demand stimulation can assist here, where DBS is delivered only when pathophysiological neural activity (linked to disease symptoms) are detected [38]. So far, such trials are limited, but there is evidence to suggest that on-demand stimulation, delivered only when elevated neural activity in the beta-frequency band is present, can improve PD motor scores beyond that of continuous high-frequency DBS, while avoiding unwanted side-effects such as speech disturbances [38]. Alternatively, fully closed-loop strategies can be employed that interact with specific neural activity [39••].

Akin to its high-frequency counterpart, low-frequency stimulation (below 100 Hz) can provide therapeutic benefits (in gait, for instance [40]), can be associated with motor degradation [41], and (at very low frequencies) has even been shown to aid cognitive performance in PD [42]. These multifarious findings reassert the dependency between stimulation parameters (such as frequency and intensity) and behavioural outcomes, and further highlight the need for intelligent forms of on-demand closed-loop stimulation that can selectively abate motor symptoms without disrupting (or perhaps even actively normalising) cognitive processes.

Unlike transcranial forms of stimulation, the electrodes employed in DBS directly innervate neural tissue, leading to a form of neuro-stimulation that is strong enough (in most cases at least) to supplant endogenous neural activity. This gives rise to the notion of an information lesion [18]. However, when the frequency (and hence the electrical energy) of stimulation is reduced, signs of brain-state dependence become more noticeable [41]. For instance, the timing of DBS pulses can be adjusted to coincide with the timing of rhythmic tremor production [43]. In Essential Tremor (ET), this leads to both entrainment and phase-specific modulation of tremor severity [44], while in PD the effects appear limited to tremor entrainment, with no significant modulation of tremor severity [43]. Indeed, similar results have been observed when regular pauses are introduced into the stimulation pulse-train, which leads to entrainment [45]. Such stimulation demonstrates that PD tremor is tolerant of intrinsic departures from its median frequency, unlike ET [39••, 46, 47]. Taken with other evidence (e.g. [48–50]), this gives rise to the question of whether PD tremor may in-fact represent a source of filtered noise. These characteristics are important for determining the most effective form of stimulation. While in ET, one could consider targeting specific phases of the tremor cycle [39••], in PD a strategy that overcomes the relative tolerance of the tremor generating system, or targets an alternative stimulation site (such as the motor cortex [51]), may be preferable.

## Transcranial Direct Current Stimulation

The field of tDCS has expanded rapidly over the past 20 years [52, 53]. With this, our understanding of the impact and interaction of tDCS with the brain, especially its pharmacological dependencies, have also developed. Novel electrode configurations have been introduced which promise to deliver stimulation in a more focal manner than ever before.

Despite these advances, recent evidence-based guidelines [54] reveal a chronic lack of sham-controlled randomised controlled tDCS trials in PD, leading to an inability of the authors to provide firm recommendations due to the small number of eligible studies. A lack of replication in protocols and experimental factors, such as the location of stimulating electrodes, further confound the problem. Nevertheless, it was concluded that there was a potential impact of anodal tDCS targeting the motor cortex on gait and motor symptoms from the small number of eligible studies [54]. These supported the notion that combining tDCS with rehabilitative strategies to enhance recovery/motor learning provided the greatest therapeutic promise [54, 55]. A further intriguing development was the finding that anodal TDCS could enhance survival and integration of dopaminergic cells in a rat model of Parkinson’s disease [56]. Thus, as in the case of rehabilitation, tDCS may make its major contribution in facilitating primary interventions, in this case cell transplantation therapies.

### Pharmacological Action and Interaction

It has long been recognised that tDCS effects are susceptible to pharmacological state [57]. Indeed, a recent review on the sensitivity of tDCS to medication state emphasised that a wide variety of drugs can reduce, enhance, or even reverse excitability effects [58•]. As such, a more thorough understanding of the underlying brain state, as influenced by medication and stimulation, is needed. This can be aided by proper documentation of medication states during experiments. Of course, physiological variability may also help to explain the broad array of sometimes contradictory evidence present in the tDCS literature (see [59]). Perhaps most importantly in relation to PD is the effect of dopamine, which produced both a dose-dependent, and receptor specific impact on tDCS excitability [60–62], with one study reporting an inverted-U shaped dose-dependent response [61]. Of course, tDCS itself results in neurochemical changes, with anodal tDCS most strongly associated with modulation of GABAergic, and also glutamatergic concentrations [57, 63, 64]. It is likely that these circular dependencies may be responsible for some paradoxical long-term responses to stimulation, including the time-dependent reversal of effects. A proper consideration of medication state is therefore a critical element when considering tDCS in trials of PD. Moreover, anatomical differences could influence and confound the stimulation effects both at the site of stimulation and on downstream nuclei. Specifically, it has been shown that axonal orientation could influence whether direct current stimulation results in excitation or inhibition [65]. However, how axonal orientation influences the net stimulation effect in complicated brain structures such as the motor cortex is still debated [66, 67].

### Field Shaping

A further consideration is the shape of the electric field generated by tCS, which dictates the area of electrical innervation in the brain. This is dependent upon the number, and geometric arrangement of stimulating electrodes, and must also take into account the complex conductivity of the brain itself. The electrodes can be positioned in a centre-surround arrangement [68], or be replaced with concentric rings [69]. However, increasing the number of electrodes generally allows greater specificity and multifocal targeting [70, 71]. These techniques are yet to be applied for the treatment of PD.

## Transcranial Alternating Current Stimulation

To date, the application of tACS on PD symptomology has remained experimental. Indeed, while the literature on tACS has expanded greatly over the past 15 years (since the reintroduction of the technique [11]), there have been only a handful of papers that have applied tACS in PD patients.

Krause et al. has so far presented the only application of beta-frequency stimulation in a PD cohort [72]. In their sham-controlled, double-blinded study, the authors assessed the impact of 20 Hz tACS on cortico-muscular coupling and motor performance in PD while patients were *ON* medication, versus a control group. Their aim was not to alleviate symptoms, but rather to assess the impact on isometric contraction and the regularity of finger tapping—since beta-frequencies are associated with bradykinesias in PD [19], and 20 Hz tACS has previously been shown to slow movement in healthy individuals [73, 74]. Stimulation attenuated beta-band cortico-muscular coupling during isometric contraction, and reduced amplitude variability during finger tapping in the PD group, but had no discernible impact on the control group. They conclude that PD may in fact be more responsive to tACS due to the altered pathophysiological brain state.

Taking a different approach, Shill et al. [75] applied high-frequency (77.5 Hz), high-amplitude (15 mA) stimulation as a prospective, offline treatment, delivering stimulation over 45 min per session for 10 days. They concluded that stimulation bore no significant benefits over placebo in UPDRS (parts I-III), anxiety, depression or sleepiness scales over the course of the experiment. Despite this, gamma frequency stimulation has been shown to speed motor responses in healthy individuals [76], and so could be further trialled as a treatment for bradykinesia.

### Adaptive Stimulation

Due to the lack of experimental data relating to open-loop stimulation, no on-demand protocols have so far been trialled. However, closed-loop stimulation has been attempted focussing on the symptom of resting tremor. Brittain et al. [51] demonstrated that tACS could partially entrain and amplitude modulate peripheral tremor as recorded by accelerometery. In this study, the peripheral movement itself was employed as a proxy for central brain activity [77], using closed-loop control to align the stimulation waveform with on-going movements in real-time. It was demonstrated that sustained closed-loop stimulation led to an order-of-magnitude increase in the effect-size of suppression of the pathological tremor over the open-loop configuration, without impacting gross motor performance. This study demonstrates the importance of temporally guided stimulation paradigms [78], especially in modalities where the strength of stimulation is weak compared to endogenous/aberrant brain activity (i.e. see [12, 79]).

The influence of tACS over the cerebellum has also been assessed in relation to PD tremor. In a cohort of mixed PD and ET patients, tACS applied between ipsilateral cerebellum and extracephalic contralateral shoulder was shown to entrain those tremors that were pliant to oscillate within a limited range of frequencies [46]. In the same study, it was shown that PD tremor displayed this pliancy to a greater extent than patients with ET, suggesting a greater tendency to entrainment. However, it should be noted that a direct comparison between PD and ET patients was not significant, possibly due to the small sample of participants in this study.

### Pharmacological Action and Interaction

When discussing tACS, the emphasis is usually on temporal alignment to maximise the impact of sub-threshold modulations in the membrane potential [78]. However, there is growing evidence that tACS is also responsible for changes in neurotransmitter concentrations and cortical plasticity. For instance, stimulation at gamma-frequencies (75 Hz in this case) has been shown to drive local GABA_A_ inhibition [80]. In addition, Guerra et al. [81] demonstrated the abolition of cholinergic short-latency afferent inhibition (SAI) during tACS at 20 Hz (separate from the phase-dependent effects that were also observed). Clearly, we must also consider the impact of alternating currents on neurochemical processes in our experimental designs, even if only to monitor and mitigate the reactive impact that these changes might cause to our neuromodulation efforts.

### Field Shaping

In both DBS and tCS, complex field shaping is made possible by developments in hardware. In tACS however, high-density electrode configurations offer the possibility to deliver not only more focal forms of stimulation, but also novel stimulation protocols. For instance, the reinforcement or disruption of synchronised neural rhythms between brain regions through in- and out-of-phase stimulation [82–85]. One downside has been the induction of steeper current gradients focussed over a smaller area that can lead to an exacerbation of sensory side effects (a particular problem when time-varying currents are employed). Indeed, perhaps the limiting factor in the delivery of tCS so far has been discomfort arising from cutaneous nociception [86].

Recently, there have been new developments in the delivery of tCS that may overcome some of these unwanted sensory side effects and, in so doing, permit the delivery of higher-intensity stimulation. The first is the suggested use of topical anaesthetics to numb sensation (as described in [86]). The second is the use of high-frequency amplitude-modulated stimulation, which delivers current beyond the response range of cutaneous receptors. This approach offers the additional benefit of reducing (or removing) stimulation artefact from the frequency range of interest, permitting simultaneous MEG or even EEG recordings to be made. The approach has been trialled with some success [87], although the knock-on impact of amplitude-modulated tACS is that the neural response to such beat frequencies is unclear; indeed, it may be that substantially stronger currents are required to induce neural modulation after signal loss due to the presumed (partial) demodulation of the stimulus waveform at the neuronal level. This is compounded by our lack of understanding about the mechanism through which demodulation occurs [88], although it possibly reflects nonlinearities in the cell membrane response [78, 87]. In support of this, triangular stimulation waveforms have been reported to modulate brain activity perhaps more effectively than sine-waves [89]. One further development is the advent of temporal interference stimulation, which applies two separate high-frequency (kHz range) stimulation waveforms simultaneously. At their intersection, an amplitude-modulated waveform is produced [90••]. This elegant solution permits considerable flexibility in field steering, and raises the exciting prospect of selectively targeting deeper brain regions. Although the technique has only been demonstrated in mice at this point, translation into human studies appears immediately feasible, although it remains to be seen whether such forms of stimulation can induce sufficient modulation in a reliable, controlled manner to offer real therapeutic prospects. One drawback of high-density arrangements is that as the focality of stimulation improves, the need for accurate placement of electrodes, and incorporation of individualised head models to provide reliable current steering becomes a necessity, significantly complicating the approach. Nevertheless, such advancements provide the opportunity to develop these techniques as either offline therapies, pre-surgical screening tools, or even as a precursor to a fully implanted epidural stimulation system.

## Direct Cortical Stimulation

Direct stimulation of the motor cortex in the treatment of PD has been trialled with varying success over the years [91]. Stimulation can be subdural, but minimally invasive epidural methods seem to be preferred due to reduced surgical risks [91]. Efficacy has been variable, but is generally considered less efficacious than DBS [92]. This is potentially due to the dependency between stimulation site (cortical or sub-cortical) and behavioural effects, and confounded by limits on stimulation intensity in order to prevent seizure onset. Stimulation efficacy could be improved by determining the optimum stimulation site according to patient specific brain connectivity in order to reveal the “sweet spot” for modulating downstream nuclei in the motor circuit [93]. This type of approaches could be important for treating disorders like PD that arise from complex network interactions. DBS is associated with greater surgical risks than epidural methods, and is not effective in all patients, especially those who prove non-responsive to dopaminergic medication [94]. Indeed, direct cortical stimulation may be a treatment option in patients who prove unresponsive to, or are otherwise contraindicative for DBS, demonstrating improvements in gait and axial symptoms [92]. Meanwhile, non-invasive forms of stimulation such as tACS has shown some promise in closed-loop forms of stimulation, but such closed-loop strategies require chronic applications, which are not currently realisable in its present form.

Could direct cortical stimulation provide a harmonious solution to some of these problems and offer a hybrid alternative therapy? Direct cortical stimulation possesses many attributes that make it a desirable intervention. It can be delivered chronically, is minimally invasive offering reduced surgical risks, and provides focal stimulation of targeted cortical regions. Does direct cortical stimulation therefore offer a convenient minimally invasive surgical option for the implementation of a chronic form of closed-loop tACS? Techniques such as temporal interference could be adapted to chronic applications across multiple electrodes [90••], offering deep epidural stimulation and targeting of midline structures. Such stimulus delivery could offer advantages over existing direct cortical stimulation, including the potential for improved efficacy. Compared to tACS, direct cortical stimulation can deliver stronger and focussed current densities. Compared with pulsatile stimulation, sine-wave modulation may reduce seizure risk due to the less acerbic nature of the stimulus.

## Common Goals and Future Directions

There are clear parallels to be drawn for the next-generation of electrical neuromodulation techniques. There is a tendency to seek greater anatomical and functional specificity. All forms have been associated with neuroplastic effects and pharmacological dependencies, and an emerging recognition that underlying brain state is an important consideration in effective neuromodulation. But these factors also overlap. Greater anatomical specificity goes hand-in-hand with the identification and preferential targeting of biological signatures of pathology, which in turn drives on-demand and closed-loop strategies. The pharmacological impact of stimulation, either by direct or indirect methods, will affect the efficacy of subsequent periods of stimulation. tDCS presently appears most suitable as an adjunct method in priming and rehabilitative training.

## Conclusions

Electrical neuromodulation remains a promising and powerful tool to interrogate neural circuits, with recent developments favouring greater anatomical and functional specificity. Techniques such as DBS are well established and have received a recent resurgence in innovation that offers new therapeutic options. Meanwhile, tCS remains a technique in its infancy, where the mechanisms of action remain unclear. As our understanding of these techniques improves, our capacity to trial novel therapeutic interventions also improves. While the surveyed approaches have shown early promise in increasing the potency and selectivity of stimulation, further clinical trials are clearly required. Continued technological developments offer the opportunity of new and innovative approaches to probe PD pathophysiology and abate the symptoms of PD.
